# Analysis of the Changes in Keratoplasty Indications and Preferred Techniques

**DOI:** 10.1371/journal.pone.0112696

**Published:** 2014-11-11

**Authors:** Stefan J. Lang, Mona Bischoff, Daniel Böhringer, Berthold Seitz, Thomas Reinhard

**Affiliations:** 1 Eye Center, Albert-Ludwigs-University of Freiburg, Freiburg, Germany; 2 Department of Ophthalmology, Saarland University Medical Center, Homburg/Saar, Germany; Cardiff University, United Kingdom

## Abstract

**Background:**

Recently, novel techniques introduced to the field of corneal surgery, e.g. Descemet membrane endothelial keratoplasty (DMEK) and corneal crosslinking, extended the therapeutic options. Additionally contact lens fitting has developed new alternatives. We herein investigated, whether these techniques have affected volume and spectrum of indications of keratoplasties in both a center more specialized in treating Fuchs’ dystrophy (center 1) and a second center that is more specialized in treating keratoconus (center 2).

**Methods:**

We retrospectively reviewed the waiting lists for indication, transplantation technique and the patients’ travel distances to the hospital at both centers.

**Results:**

We reviewed a total of 3778 procedures. Fuchs’ dystrophy increased at center 1 from 17% (42) to 44% (150) and from 13% (27) to 23% (62) at center 2. In center 1, DMEK increased from zero percent in 2010 to 51% in 2013. In center 2, DMEK was not performed until 2013. The percentage of patients with keratoconus slightly decreased from 15% (36) in 2009 vs. 12% (40) in 2013 in center 1. The respective percentages in center 2 were 28% (57) and 19% (51). In both centers, the patients’ travel distances increased.

**Conclusions:**

The results from center 1 suggest that DMEK might increase the total number of keratoplasties. The increase in travel distance suggests that this cannot be fully attributed to recruiting the less advanced patients from the hospital proximity. The increase is rather due to more referrals from other regions. The decrease of keratoconus patients in both centers is surprising and may be attributed to optimized contact lens fitting or even to the effect corneal crosslinking procedure.

## Introduction

Since the first successful corneal transplantation by Zirm in 1906 [1] the field of keratoplasty has experienced a continuous change and expansion in the spectrum of treated corneal pathologies. Inflammatory corneal diseases used to be one of the most important indications in the mid-20^th^ century and were then replaced by graft failure and bullous keratopathy [2] with and without intraocular lenses [3]. From the 1980s on, bullous keratopathy remained an important indication for keratoplasty [4], [5]. During the 1990s however, in Germany the treatment of keratoconus and Fuchs’ dystrophy gained importance and was among the leading indication for keratoplasty [6], [7]. From 2000 on, both keratoconus and Fuchs’ dystrophy were among the most important indications [8]–[10]. Also in other regions graft failure, keratoconus and Fuchs’ endothelial dystrophy were rated among the frequent indications [5], [11].

The first anterior lamellar keratoplasty was performed at the end of the 19th century [12]. The first references on lamellar keratoplasty date back from the 1950s [13], [14]. The lamellar techniques were reintroduced beginning in 1998 and widely used recently [15], [16]. They became the treatment of choice in corneal diseases involving only certain layers of the cornea [17]. Different techniques of endothelial keratoplasty were introduced for treating Fuchs’ dystrophy. Descemet stripping with endothelial keratoplasty (DSEK) [18], Descemet stripping automated endothelial keratoplasty (DSAEK) [19] and Descemet membrane endothelial keratoplasty (DMEK) [20] are widely used in treatment of Fuchs’ dystrophy. Lamellar techniques are also available for the treatment of keratoconus [21]. Especially the deep anterior lamellar keratoplasty (DALK) appears to be most promising [22], [23].

In the treatment of keratoconus conventional penetrating keratoplasty used to be the treatment of choice over more than 60 years. There are several methods of trephination for the penetrating keratoplasty, including manual trephination with free-hand trephine blades or suction fixated trephine systems. Laser assisted methods trephination include the excimer laser keratoplasty [24]–[26] or the femtosecond laser assisted keratoplasty [27], [28]. These techniques are used in different corneal diseases including keratoconus [29]–[31].

In our opinion the new introduction of surgical techniques might have an impact on the indications for keratoplasty depending on the preferably used techniques and specializations of a center. The lamellar techniques for example might replace the conventional keratoplasty in some indications like Fuchs’ dystrophy. Another question is if an eye bank can maintain the demand for corneal transplantation in its area or if the demand decreases over time due to loss of appropriate patients, either due to successful surgery or change in the therapeutic approach. The use of contact lenses and corneal crosslinking for instance might supersede the conventional keratoplasty as treatment of choice for keratoconus. Possible compensatory measures might include recruitment of new patients with a higher mobility from more distant areas, or a change in the surgical spectrum with a focus on a different group of patients.

The purpose of this study was to investigate the volume and spectrum of indications for keratoplasties in a center more specialized in treating Fuchs’ dystrophy (center 1) and a second center that is more specialized in treating keratoconus (center 2). In center 1 lamellar keratoplasty with DSAEK and DMEK was adopted early for the treatment of Fuchs’ Dystrophie. Center 2 is specialized in excimer keratoplasty, especially for treatment of keratoconus. Air-line distance between both centers is 94 miles. To determine the mobility of the patients we also registered the patients travel distance from their homes to the respective center.

## Methods

We retrospectively reviewed the eye bank data of 2 centers specialized in the treatment of corneal disorders with penetrating or lamellar keratoplasty. Center 1 is more specialized in the treatment of Fuchs’ dystrophy, center 2 is more specialized in the treatment of keratoconus. Ethics Committee approval was obtained at Albert-Ludwigs-University of Freiburg and Saarland University Medical Center. Prior to analysis, all patient records and information were anonymized and de-identified. A descriptive analysis of the data was performed for each year with regard to the indication for the keratoplasty and the surgical technique and is presented as flowcharts and box plots. We also analyzed the travel distance from the patient’s home to the hospital and calculated the air plane distance on the basis of the postcode. Unfortunately foreign patients could not be included in the travel distance analysis.

## Results

**Figure 1:**
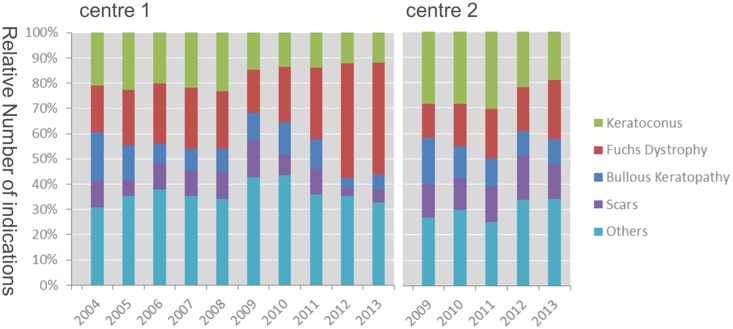
Indication for transplantation in both centers.

A total of 3778 surgical procedures were analyzed in our study. In center 1 the data from 2004 to 2013 and in center 2 the data from 2009 to 2013 were available for analysis. The number of surgical procedures per year steadily rose in both centers. Center 1 showed an increase from 182 procedures in 2004 to 335 procedures in 2013. In center 2 there was an increase from 202 in 2009 to 267 procedures in 2013. The number of patients with Fuchs’ dystrophy in center 1 remained roughly stable between 2004 (18%, 34) and 2010 (21%, 59). Thereafter, this subgroup increased dramatically: 2011 (28%, 74) to 2012 (45%, 147) and 2013 (45%, 150). The increase was less pronounced in center 2: 13% (27) in 2009 to 23% (62) in 2013. The percentage of patients with keratoconus was stable during 2004 (21%, 38) to 2009 (23%, 71) in center 1. Thereafter this percentage decreased down to 12% (40) in 2013. In center 2 28% (57) of indications was keratoconus in 2009 and 19% (51) in 2013. The number of patients with bullous keratopathy showed upturns and downturns over the years reaching their highest number in 2004 in center 1 (33%, 35) and in 2009 in center 2 (18%, 37). The lowest number was in 2013 in center 2 (10%, 26). Corneal scars also fluctuated throughout the years. The highest percentage was 17% (39) in center 2 in 2012. The lowest count was in center 1 in 2012 (3%, 10). Other indications comprised 25% to 45%. All percentages are detailed in Figure 1.

**Figure 2:**
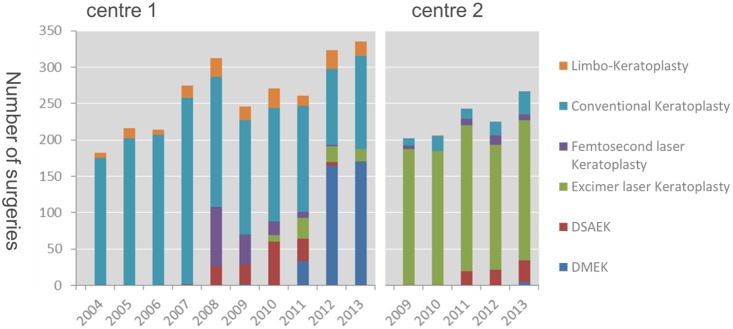
Total number of keratoplasties in both centers.

Conventional penetrating keratoplasty was the surgical method of choice in center 1 until 2007 (93%) for all indications. After that the number decreased to 38% in 2013. In 2008 DSAEK (8%) and penetrating femtolaser assisted keratoplasty (26%) gained importance. The number of DSAEK surgeries in center 1 increased until 2010 (22%, 60). In 2011 DMEK began to replace DSAEK completely. DMEK peaked in 2013 with 51% (170). The femtolaser assisted keratoplasty was abandoned in 2013 in center 1. Penetrating excimer laser keratoplasty was introduced in 2010 in center 1 (3%, 9). The number increased to 11% (29) in 2011 and then remained stable at about 5% (2013). Allogenic limbo-keratoplasty provided a maximum of 10% of keratoplasties in all indications. The total number of surgeries per year can be seen in Figure 2.

In center 2 excimer laser keratoplasty has been predominantly used for penetrating keratoplasty. In 2009 92% of procedures were excimer laser keratoplasties. The percentage decreased to 72% in 2013. The total number remained almost the same (187 in 2009, 193 in 2013). The percentage of conventional keratoplasties reached a maximum in 2013 with 12% (32). DSAEK was started in 2011 and accounted for 8% of procedures. In 2013 11% (30) of surgeries were DSAEK. DMEK was not performed before 2013. Femtosecondlaser assisted keratoplasty was only done in a small number of cases.

**Figure 3:**
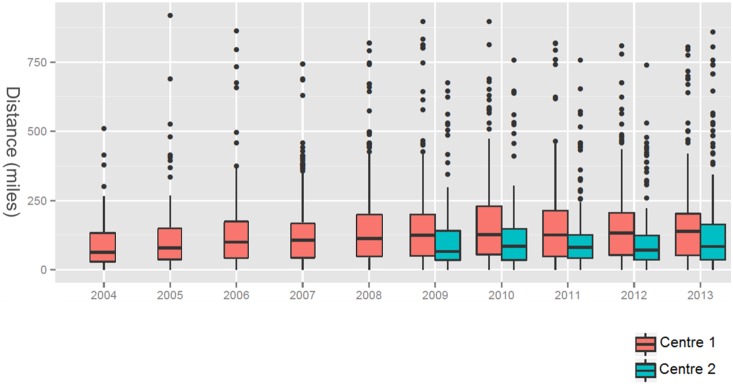
Mean airplane distance from the patients’ homes to the hospital. Foreign patients could not be included in the distance evaluation.

The patients’ travel distances increased in both centers (Figure 3). This increase was more pronounced in center 1 where the mean linear distance from the patients’ homes to the hospital increased from 55 miles in 2003 to 146 miles in 2013 (center 2: 65 miles in 2009 to 80 miles in 2013).

## Discussion

In both centers keratoconus, Fuchs’ dystrophy and bullous keratopathy used to be more or less equally presented. This is in accordance with the results of previous data [6], [7]. In the recent years both centers showed a decline in the relative amount of bullous keratopathy cases and of keratoconus patients. This is concordant with the results of other studies, that also showed a decline in bullous keratopathy cases [7], [11]. The decline in bullous keratopathy cases may be attributed to the less frequent application of anterior chamber intraocular lenses and the restriction of cataract surgery to experienced surgeons in Germany. Therefore, in other regions the bullous keratopathy may still be a major factor leading to a keratoplasty as was reported in the past years [5], [11], [17].

The decline in keratoconus patients is surprising since from 1992 until 2010 several authors reported keratoconus among the leading indications for keratoplasty [6]–[8]. In center 1 the total number of keratoconus patients also fell whereas in center 2 the total number of keratoconus patients stayed almost the same but did not parallel the total number of keratoplasties. This may be attributed to optimized contact lens fitting or the widespread application of corneal crosslinking.

Fuchs’ dystrophy showed an increase in both centers and was the major indication for keratoplasty in 2013. From the 1980s, where Fuchs’ dystrophy ranged among the rare indications for keratoplasty [4], [5], there was an increase in Fuchs’ dystrophy in many studies. However unitl now, it did not provide the major indication for keratoplasty [8], [11].

Both centers showed a decline in the relative number of penetrating keratoplasies while posterior lamellar keratoplasties increased immensely.

Our results show, that a new technique such as DMEK may replace the penetrating keratoplasty as most used technique in a single center. Even though there is a decline in all other major indications like keratoconus or bullous keratopathy the total number of transplantations increased. A reason is that a vast group of patients, which was not suitable for penetrating keratoplasty, now appears to be eligible to transplantation via DMEK. This can be attributed to a recruitment of less advanced patients. It is unclear whether one technique (DMEK or DSAEK) has a crucial advantage over the other [32] or which method of graft preparation is superior [33] or the influence of the selection of the right donor tissue [34] is still subject to current research. In addition, the question is still open for the discussion, what is the appropriate visual acuity Fuchs’ dystrophy patients should be offered posterior lamellar keratoplasty.

Another compensatory measure to maintain a steady demand is a recruitment of patients that live further away from the hospital. The increasing travel distance shows, that the patient mobility is high enough to reach a center specialized in their kind of pathology.

In conclusion the results show that DMEK increases the total number of keratoplasties. This cannot be fully attributed to recruiting the less advanced patients from the proximity of the hospital but rather due to more referrals from other regions. The decrease of keratoconus patients in both centers is surprising and may be attributed to optimized contact lens fitting or to the stabilization effect of corneal crosslinking.
